# Unveiling absent inferor vena cava in young patients: Case reports and clinical insights

**DOI:** 10.1016/j.ijscr.2024.110258

**Published:** 2024-09-07

**Authors:** Niki Tadayon, Dorsa Najari, Meisam Refaei, Mohsen Sheikhzadeh, Masoud Babaei, Mohammad Moein Mirhosseini

**Affiliations:** Division of Vascular and Endovascular Surgery, Department of Surgery, Shohada-Tajrish Medical Center, Shahid Beheshti University of Medical Sciences, Tehran, Iran

**Keywords:** Deep vein thrombosis (DVT), IVC embryology, Collateral veins, Agenesis, Inferior vena cava, Case report

## Abstract

**Introduction:**

Inferior vena cava agenesis (IVCA), a rare congenital anomaly, contributes to approximately 5 % of deep venous thrombosis (DVT) cases lacking other risk factors. It can lead to chronic venous insufficiency and DVT when collateral circulation is insufficient, presenting diagnostic challenges due to its rarity.

**Case presentation:**

We present two cases of Absent IVC (AIVC) in young males. Case 1: a 22-year-old developed bilateral lower limb DVT post-appendectomy. Imaging revealed AIVC with azygos continuation. Treatment included Heparin and Rivaroxaban, achieving symptom resolution. Case 2: a 41-year-old with recurrent DVT and chronic venous insufficiency was diagnosed with AIVC via venography. Managed with warfarin and compression therapy, his symptoms stabilized.

**Clinical discussion:**

These cases underscore the importance of recognizing AIVC in young patients presenting with unexplained DVT. Diagnosis often requires advanced imaging techniques like CT venography. Management typically involves long-term anticoagulation and compression therapy to mitigate the risk of recurrence and chronic venous complications.

**Conclusion:**

Early identification of AIVC in young adults presenting with recurrent DVT is essential for appropriate management and prevention of long-term complications.

## Highlights

• This article presents two cases of Inferior Vena Cava Agenesis (IVCA), a rare congenital anomaly that accounts for about 5 % of unexplained DVT cases.
• The first case involves a 22-year-old male with bilateral DVT following appendectomy, and the second a 41-year-old male with recurrent DVT and chronic venous insufficiency. Both underscore the need to consider IVCA in unexplained DVT.
• Early identification of IVCA in young adults with DVT is crucial for effective management and prevention of complications.
• The article highlights the importance of personalized treatment strategies, including lifelong anticoagulation and compression therapy, to manage recurrence and long-term venous issues.

## 1 Introduction

Inferior vena cava agenesis (IVCA) is thought to be responsible for 5 % of all deep venous thrombosis (DVT) events with no other risk factors [1]. Although it normally causes no symptoms, it can occasionally appear with advanced stages of chronic venous insufficiency [2]. CT scans reveal particular aspects of Absent IVC (AIVC): when a portion of the IVC is absent, the iliac veins drain into larger ascending lumbar veins. Extensive collateral blood flow occurs, particularly through the prominent azygos and hemiazygos systems, as well as veins along the spine and the front of the abdominal wall. These symptoms are most common when there is insufficient collateral blood flow, which causes progressive venous stasis and can develop to unilateral or bilateral DVT.

However, due to the rarity of this diagnosis, it often goes undetected. Herein, we report two consecutive young patients, both finally diagnosed with AIVC, but presented with different scenarios.

The work in this case presentation has been reported in line with the SCARE criteria, and the patients provided informed consent [3].

## 2 Case report 1

A 22-year-old male presented with bilateral lower limb pain and swelling. Two weeks prior to this presentation, he underwent open appendectomy at another hospital. His symptoms (pain and swelling), began two days post-operation. At that hospital, he was diagnosed with DVT. Doppler sonography revealed complete thrombosis of the left and right common femoral vein (CFV), superficial femoral vein (SFV), saphenofemoral junction (SFJ), and popliteal veins. Consequently, he was started on Warfarin and a Heparin (UFH) intra venous infusion. However, due to a lack of improvement after one week, he sought care at our facility.

The patient denied any additional trauma or recent travel. Physical examination revealed swelling along the lower limbs, extending from the foot to the thigh. The limbs were tense, although distal neurovascular function remained intact. Based on the initial clinical evaluation, a diagnosis of DVT was made. To further assess his condition, the patient underwent Doppler sonography, CT venography, and magnetic resonance venography (MRV).

**Fig. 1:**
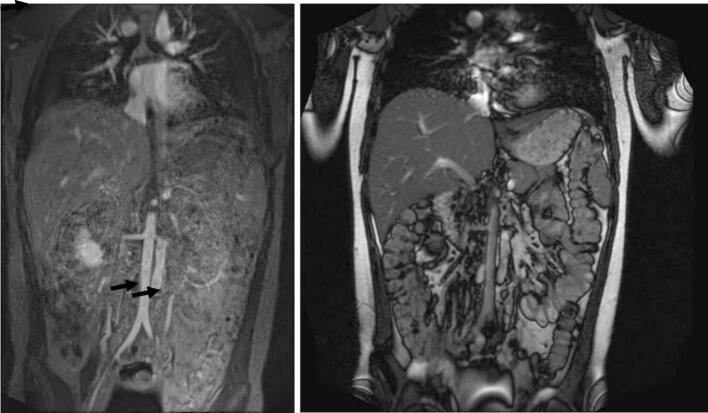
Magnetic resonance venography of case 1. On the right, aorta. On the left, abnormal inferior vena cava.

CT venography reported an interruption in the inferior vena cava (IVC) with azygos continuation, while both renal veins appeared normal. Abdominal MR venography indicated that the supradiaphragmatic IVC was normal (Fig. 1). However, on the right side of the abdominal aorta, the IVC was not properly visualized, and azygos continuation was noted. Laboratory results were normal.

The patient was then treated with Heparin (UFH) was administered as an intravenous infusion, starting with a loading dose of 100 units/kg, followed by a maintenance infusion of 1000 units per hour, with the goal of achieving a PTT 2–3 times higher than the normal upper limit. Consequently, lower limbs elevation was done. He reported no complaints after one day of admission. He was subsequently discharged with a prescription for Rivaroxaban 15 mg twice daily. Two weeks post-discharge, he was revisited. All his symptoms were relieved. His drug dosage was changed to 20 mg daily, and compression therapy was recommended.

## 3 Case report 2

A 41-year-old male presented with a history of recurrent DVT, varicose veins, and chronic venous insufficiency affecting both lower limbs and the genital area. The patient reported experiencing two separate episodes of DVT. He also mentioned a previous history of venoplasty for varicose veins two years ago, though no documentation was available.

Clinical examination revealed varices from distal to proximal in both limbs, worsening distally. His medication history included the use of warfarin, which was transitioned to heparin using bridge therapy. Heparin was administered with a loading dose of 100 units/kg, followed by a maintenance infusion of 1000 units/h. Warfarin was initially given as one tablet daily before the transition. He denied any other risk factors.

**Fig. 2:**
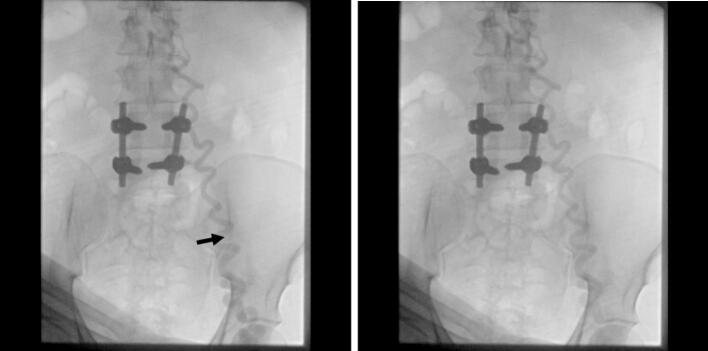
Collateral vein washing out contrast, indicating severe turtosity venography in the operating room, case 2.

Due to an unreliable history, worsening varicose veins and our clinical evaluation, he underwent elective venography in the operating room. During the procedure, unsuccessful cannulation attempts revealed tortuosity of the superficial vein. Consequently, under ultrasonography guidance, venography was performed via the left femoral vein, and proper sheath placement was achieved, leading only to washout of the collateral veins and raising suspicion of IVC malformations (Fig. 2).

**Fig. 3:**
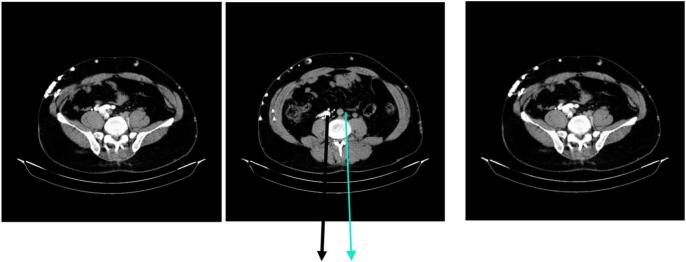
Computed tomography scans of case 2, green arrow indicating aorta, as the black one indicates an abnormal collateral vein. (For interpretation of the references to color in this figure legend, the reader is referred to the web version of this article.)

For a better diagnosis, we decided to perform Computed tomography Venography Scan (CTV Scan). The CTV detected an absence of the inferior vena cava (IVC) from the infrarenal area. Additionally, there was involvement of pelvic veins and collateral varicosities in the abdomen and lower limbs due to venous return disorder. The intrahepatic IVC was visualized up to the anatomical drainage site of the renal veins (Fig. 3).

Laboratory data were within expected ranges.

He was eventually discharged with a prescription for warfarin, alongside compression therapy, aiming to maintain an INR level of 2–3, and a diagnosis of AIVC.

## 4 Discussion

Congenital IVC abnormalities are considered a rare condition, with a prevalence estimated at 0.3–0.6 % in healthy individuals. However, they are most likely related with a persistent risk of thrombosis throughout one's life. For example, it has been discovered that people with congenital IVC anomalies usually develop DVT at a young age. In contrast, DVT is highly rare in the overall healthy population of young patients, with a tenfold lower incidence among those aged 20 to 40 [[4], [5], [6]]. Both of our patients somehow belonged to this age bracket. DVT in AIVC was more common in males, as reported in most other studies, including ours.

AIVC can easily be missed. There are several diagnostic procedures available for detecting AIVC.

Venous duplex ultrasound imaging is the most straightforward method for patients with suspected lower extremities DVT or symptoms of chronic venous insufficiency [2]. However, venous duplex imaging may not be sufficient to detect AIVC or other IVC abnormalities. Given our situations, particularly the second one, despite previous studies, no conclusive diagnosis was made. Current NICE (National Institute for Health and Care Excellence) guidelines for DVT emphasize an organized approach to diagnosis that includes the Wells Score, D-dimer tests, and ultrasonography. However, because inferior vena cava aneurysms are rarely evident on ultrasonography, their diagnosis is frequently incidental or occurs if it is anticipated previously [7].

It is believed that most cases remain asymptomatic due to the extensive collateral venous network in the abdomen and lower extremities. However, compared to the general population, there will always be some degree of venous stasis despite the presence of these collateral pathways [8]. Our two cases, while sharing the diagnosis of AIVC, exhibited distinct clinical presentations. The first case involved a 22-year-old male who developed DVT shortly after undergoing an appendectomy. This incident of DVT, likely provoked by the surgical procedure, highlighted the risk of chronic venous insufficiency in the near future, if left unrecognized. It is imperative to consider congenital anomalies such as AIVC in young patients presenting with DVT following minor events like minor surgeries. This simple reminder for clinicians can help prevent patients from facing future risks of painful chronic venous insufficiency episodes, venous malformations, varicose veins, and the need for extensive medical evaluations and procedures.

Regarding treatment, current approaches are mostly conservative and include anticoagulation, compression stockings, thrombolysis, and thrombectomy or angioplasty, similar to other DVT patients. There are no specific recommendations for these patients, and treatment decisions are typically made on a case-by-case basis by the clinician [9]. A recent systematic review, the largest population study available, included 216 patients with IVC atresia (covering studies up to 2020) [10]. It found that 66.5 % of patients received anticoagulation therapy, with 65.2 % treated with anticoagulation alone. The duration of therapy remains controversial, with 53.2 % receiving long-term or indefinite treatment. Endovascular venous reconstruction was successfully performed in a subset of patients, with a significant portion showing symptom improvement. Although some required reintervention, the outcomes support endovascular recanalization as a viable and less invasive alternative to open surgery. Surgical intervention, whether endovascular or open, should be considered for patients with advanced venous insufficiency or those who do not respond to anticoagulation therapy.

It is essential to ensure regular follow-ups for patients diagnosed with absent IVC, as recurrent DVTs can occur, as seen in our second patient, who experienced recurrence while on oral Warfarin, later found to be at a sub-therapeutic dose. Consistent monitoring is equally important for patients who undergo more invasive procedures, to minimize the risk of complications such as stent migration. Finally, it is safe to say that prolonged anticoagulation, perhaps even life-long, is usually beneficial to these patients along with elastic stockings.

## 5 Conclusion

Absent IVC is a notable but often overlooked cause of recurrent DVT, particularly in younger males under 40 who present with unexplained venous issues. Recognizing this condition can significantly benefit both patients and healthcare systems by facilitating early diagnosis and targeted management, thus preventing future complications. While lifelong anticoagulation may be beneficial, its necessity remains a topic of ongoing debate and should be considered on a case-by-case basis. Enhanced awareness and careful evaluation are essential for optimizing patient outcomes and guiding appropriate treatment strategies.

## Declaration of competing interest

No conflict of interest.
